# Review of “*Stereochemistry Workbook, 191 Problems and Solutions*” by K.-H. Hellwich and C. D. Siebert

**DOI:** 10.3762/bjoc.5.38

**Published:** 2009-08-05

**Authors:** Roald Hoffmann

**Affiliations:** 1 Dept. of Chemistry and Chemical Biology, Cornell University, Baker Laboratory, Ithaca NY 14853, USA

Hellwich, K.-H.; Siebert, C. D. *Stereochemistry Workbook*, *191 Problems and Solutions*. transl., A. D. Dunn Springer-Verlag: Berlin, Heidelberg, 2006. XI, 198 pages, ISBN 978-3-540-32911-4

The late Edgar Heilbronner, one of the great physical and organic chemists of the 20^th^ century, had a particularly effective lecture. He started it as follows: “You know pretty much everything I am going to say. So let us begin with the questions.” And up on the screen flashed the first question. The lecture continued in the same vein; Heilbronner’s carefully structured answers to just the right questions led to a fascinating seminar, and a learning experience.

I thought of Edgar Heilbronner’s lecture as I read the Hellwich and Siebert “Stereochemistry Workbook.” In it are 191 graduated problems, along with succinct and clear solutions. One has to know a little stereochemical lore to begin with, for instance the priority rules of the CIP system. But otherwise everything is taught, in exemplary fashion, just through the progression of questions and answers. Complexities of naming coordination compounds, and dealing with chirality planes and axes are not avoided. The examples grow increasingly challenging.

The success of this small volume lies in the fact it has a hidden narrative structure. Complexity is introduced in stages; solutions to the problems are attainable goals. One is drawn in, it is fun to go on. And the book is decorated with fascinating short stories of pharmaceutical design and utility. We have here a rare instance of a well-designed workbook that constructs a learning experience better than that found in most textbooks.

What more would I want to see in it? The ordinary (of reasonable Lewis structures, normal bond lengths, moderate temperature [*T* less than ~100 °C]) which is the province of the beautiful compounds that are discussed in this book, needs to be defined. The ordinary is not diminished by what constrains it, for the fantastic variety the normal world generates suffices. But it is good to be aware of just what the assumptions are.

Let me give an example. In one problem, a set of isomers of a bicyclic compound (cyclothiazide) is eliminated from discussion because a bridge can be formed only by *cis* fusion. In another example, two isomers of a [CoCl_2_(H_2_NNH_2_)(PPh_3_)_2_] complex are said to be not possible, those where the H_2_NNH_2_ would bridge *trans*-positions on the octahedral metal. Indeed, both statements make sense, but it would be interesting to mention why they are true (strain, the long N–N bond necessary). And how it is in the nature of chemists to subvert the impossible, and what ingenious molecules might do if there is no option but the energetically costly impossible.

“Stereochemistry Workbook,” one of the most effective pedagogical texts I’ve encountered, re-emphasizes the fundamental role of geometry in chemistry. Molecules have shapes. The logic of isomerism, in all its manifestations, the life-and-death-determining necessity of describing three-dimensional geometry, our representational struggles to do so – these define the beauty of chemistry.

